# Gender bias in fetal malformations: A cross-sectional study in Asian populations

**DOI:** 10.3389/fendo.2023.1146689

**Published:** 2023-03-30

**Authors:** Meixiang Zhang, Yingchun Su, Ying-pu Sun

**Affiliations:** ^1^ Center for Reproductive Medicine, First Affiliated Hospital of Zhengzhou University, Zhengzhou, China; ^2^ Henan Key Laboratory of Reproduction and Genetics, First Affiliated Hospital of Zhengzhou University, Zhengzhou, Henan, China

**Keywords:** sex ratio, induced abortion, fetal malformation, cross-sectional study, single nucleotide polymorphism (SNP) array, gender bias

## Abstract

**Objectives:**

The aim of this study was to detect any gender bias in fetal malformation cases.

**Design:**

This study was a cross-sectional, quantitative survey.

**Subjects:**

Overall, 1,661 Asian fetal malformation cases involving induced abortions in the obstetrics department of the first Affiliated Hospital of Zhengzhou University from 2012 to 2021 were included.

**Main outcome measures:**

Measurements of ultrasound detectable structural malformations were classified into 13 subtypes. Karyotyping, single nucleotide polymorphism (SNP) array, or sequencing diagnosis of these fetus was also included in the outcome measures.

**Results:**

The sex ratio (male/female) of all malformation types was 1.446. Cardiopulmonary had the highest proportion of all malformation types with 28%. Diaphragmatic hernia, omphalocele, gastroschisis, nuchal translucency (NT), and Multy malformations had significantly higher proportions of males (*p* < 0.05). Digestive system malformations had a significantly higher proportion of females (*p* < 0.05). Maternal age was associated with genetic factors (*r* = 0.953, *p* < 0.001) and inversely associated with brain malformations (*r* = −0.570, *p* = 0.002). More males were found with trisomy 21, trisomy 18, and monogenetic diseases, while duplications, deletions, and uniparental disomy (UPD) had similar sex ratios between males and females, but not statistically significant.

**Conclusion:**

Sex differences are common with fetal malformations, with higher proportions of males. Genetic testing has been proposed to account for these differences.

## Introduction

A slightly higher proportion of males exist in the human population, regardless of race and region (1). However, data strongly suggest that human male gametes carry the same proportion of X and Y chromosomes by meiosis (1). It is unclear whether fetal loss during pregnancy is responsible for the slightly higher proportion of male births. Several studies have attempted to explain this phenomenon, such as placental dysfunction (2) or hormone levels (3). Yet, neither appeared to provide sufficient evidence. In recent years, with the development of sequencing and microarray technologies, more work has focused on the causes of sex ratio alternation at the genetic level. Studies have shown that certain mutations also cause gender bias (4), but which gender is disproportionate for various malformation types remains unclear. In this study, we collected 10 years of data to analyze the sex ratio with various malformations and their relationship with maternal age. We also examined the sex ratio of fetuses with various gene-related abnormalities after genetic diagnoses were performed. A better understanding of sexual predominance can help with diagnosis and predicting risk in patients, as well as affect decisions on diagnostic testing.

## Materials and methods

This study was conducted with the approval of the Institutional Review Board, and informed consent was obtained. This study collected cases of fetal malformation from January 2012 to December 2021 from the First Affiliated Hospital of Zhengzhou University. Data were collected from Hospital Information System (HIS) with the following inclusion criteria: (1) over 12 weeks of gestation; (2) ultrasound diagnosed fetal malformations or genetic test [karyotyping, single nucleotide polymorphism (SNP) array, or sequencing] abnormal fetus; (3) terminated the pregnancy in our hospital; (4) maternal age ≥ 20 years old and ≤ 45 years old the gender of the fetus was described clearly in the medical record. In addition, exclusion criteria: (1) diagnosed with fetal malformation but terminated the pregnancy in another medical institution; (2) diagnosed with fetal malformation but continued pregnancy; (3) diagnosed with fetal malformation, but combined with maternal disease, induced abortion from maternal disease; (4) cases with incomplete medical records. Except for monogenic diseases, genetic testing of amniotic fluid and induced fetal tissue following an abnormality detected by ultrasound is based on voluntary principles. The materials mainly used for genetic testing include induced fetal tissue (fetal skin), amniotic fluid, and fetal villi. The methods of testing are mainly gene sequencing, used primarily for monogenic diseases, and microarray-based comparative genomic hybridization (aCGH) or SNP array, used mainly for detecting aneuploidy, copy number variations, uniparental disomy (UPD), and polyploidy. Note that some monogenic diseases may be detected incidentally during gene sequencing. Using the inclusion and exclusion criteria, 1,661 patients were screened for analysis. Among them, 1,007 of 1,661 cases contained only one type of malformation, which were applied into the percentage calculation of malformation types. Other analyses included all cases with solitary and multisystem malformations. Sex ratio and maternal age relative calculations used the number of cases that included the specific kind of malformation to reflect the relationship between these factors and such deformities.

Numerical variables are presented as mean ± standard deviation, and categorical variables are presented as frequencies. Associated with maternal age and the ratio of malformations was revealed by Spearman’s correlations analysis. Differences between categorical variables were compared *via* chi-squared tests. All analyses were performed using SPSS 22.0 software (IBM Corp., Armonk, NY, USA) and R 3.6.3. Differences were considered significant at *P* < 0.05.

## Results

A total of 1,661 fetal malformation cases were included in the analysis. All kinds of structural malformations were classified into 13 types: cardiopulmonary dysplasia (cardiopulmonary); cleft lip and palate (CLP), nuchal translucency (NT) abnormalities with or without other deformities (NT/Multy); brain-related malformations and arachnoid cysts (brain); urinary or reproductive system malformations (urogenital system); diaphragmatic hernia, omphalocele, gastroschisis (chest and abdomen); limbs, bones, hands, and feet abnormalities (limbs/bones); spine and vertebral body related deformities (spine); hydrocephalus (hydrocephalus); teratoma (teratoma); digestive system abnormalities (digestive system); pleural effusion (pleural effusion); Extraembryonic coelom (extraembryonic coelom). Excluding structural malformations, karyotyping, SNP arrays, or sequencing data that showed abnormalities (genetic factor) were also analyzed in our study. Overall, 1,007 fetuses had a single type of malformation, 616 fetuses had more than one type of malformation, and the other 38 fetuses were unable to be classified into the 13 different types.

### Malformation types and sex ratio

**Figure 1:**
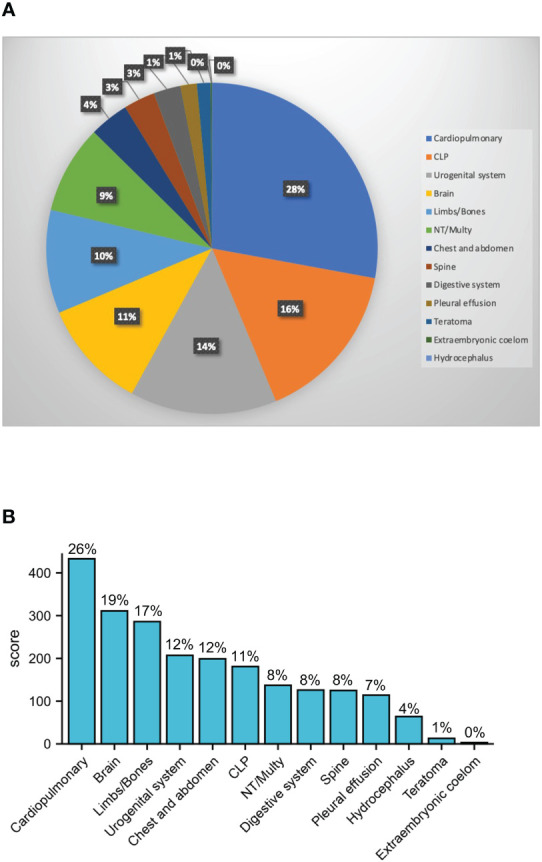
Sex ratio of 13 types of structural malformations. **(A)** Pie chart for the ratio of 13 types 1007 solitary malformations; **(B)** case number of 13 types of all 1632 structural malformation, the percentage shows malformations that contains such deformities (cardiopulmonary: cardiopulmonary dysplasia; CLP: cleft lip and palate; NT: nuchal translucency; Multy: abnormalities with or without other deformities; Brain: brain-related malformations and arachnoid cysts; urogenital system: urinary or reproductive system malformations; chest and abdomen: diaphragmatic hernia, omphalocele, gastroschisis; limbs/bones: limbs, bones, hands, and feet abnormalities; spine: spine and vertebral body related deformities; digestive system: digestive system abnormalities).

Among the 1,661 structural malformation cases, the maternal age ranged from 20 to 45 with an average of 29.5 years old, 872 were male and 603 were female, while sex was unable to be detected in the other 186 cases. The overall male to female sex ratio of all malformation fetuses was 1.446. We derived the proportions of the 13 types of structural malformations by classifying the 1,007 solitary malformations. Cardiopulmonary had the highest proportion with 28%. CLP, urogenital system, brain, limbs/bones, and NT/Multy were more than 5% (**Figure 1A**). Because about 40% of malformations included multiple types, we calculated the proportion of cases with various malformations. If the fetus had this type of abnormality, she/he would be included into the malformation type. The results showed that cardiopulmonary abnormalities were also the most common type of malformation (26%), followed by brain and limb/bone developmental abnormalities (**Figure 1B**). We found that the sex ratios of the cardiopulmonary, brain, limbs/bones, urogenital system, CLP, spine, pleural effusion, hydrocephalus, teratoma, and extraembryonic coelom classifications were similar to the overall sex ratio. However, the chest and abdomen and NT/Multy malformation classifications had more males with sex ratios of 2.240 and 2.583, respectively. Digestive system malformations had more female fetuses with a sex ratio of 0.926 (**Table 1**).

**Table 1:** Sex ratio of 13 types of structural malformations (Chi-squared tests applied to categorical variables, corrected *X^2^*), P < 0.05 (bold)).

	Male	Female	Unknown or others	Total	Male/female ratio	Corrected *X2*	*P*
Cardiopulmonary	235	162	36	433	1.451	0.001	0.976
Genetic factor	227	148	54	429	1.534	0.192	0.660
Brain	168	117	26	311	1.436	0.000	0.990
Limbs/bones	140	109	37	286	1.284	0.621	0.431
Urogenital system	102	82	23	207	1.244	0.770	0.380
Chest and abdomen	112	50	37	199	2.240	5.698	**0.017**
CLP	103	66	12	181	1.561	0.141	0.707
NT/Multy	62	24	51	137	2.583	5.165	**0.023**
Digestive system	50	54	22	126	0.926	4.431	**0.035**
Spine	58	46	21	125	1.261	0.322	0.570
Pleural effusion	51	45	18	114	1.133	1.100	0.294
Hydrocephalus	39	23	2	64	1.696	0.214	0.644
Teratoma	7	6	0	13	1.167	0.010	0.919
Extraembryonic coelom	1	1	1	3	1.000	0.209	0.647
Total	872	603	186	1661	1.446		

### Malformation types and maternal age

**Figure 2:**
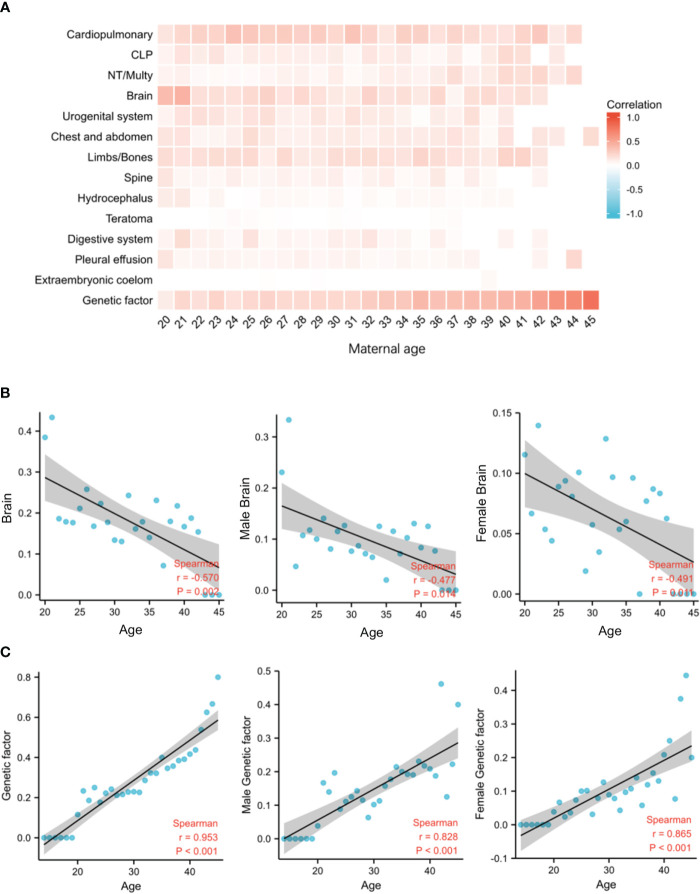
The relationship between malformations and maternal age. **(A)** Ratio of 13 types of structural malformations and maternal age. **(B)** The relationship between Brain-related malformations and arachnoid cysts (brain) and maternal age (total: *r* = 0.570, *p* = 0.002; male: *r* = 0.477, *p* = 0.014; female: *r* = 0.491, *p* = 0.011). **(C)** The relationship between genetic factor and maternal age (total: *r* = 0.95*3*, *p* < 0.001; male: *r* = 0.828, *p* < 0.001; female: *r* = 0.865, *p* < 0.001).

We then analyzed the relationship between each malformation type and maternal age. Genetic factors were also included in the analysis. The data showed that most structural malformations were unrelated to maternal age, except for brain malformations and genetic factors for both male and female fetuses (**Figure 2A**). The incidence of brain malformations was inversely associated with maternal age, with correlation coefficients of 0.570 (total, *p* = 0.002), 0.477 (male, *p* = 0.014), and 0.491 (female, *p* = 0.011) (**Figure 2B**). However, the incidence of genetic factors was positively correlated with maternal age, with correlation coefficients of 0.953 (total, *p* < 0.001), 0.828 (male, *p* < 0.001), and 0.865 (female, *p* < 0.001) (**Figure 2C**). Other malformations not associated with maternal age are also listed in **Supplementary Figure S1**.

### Genetic testing for malformed fetuses

**Figure 3:**
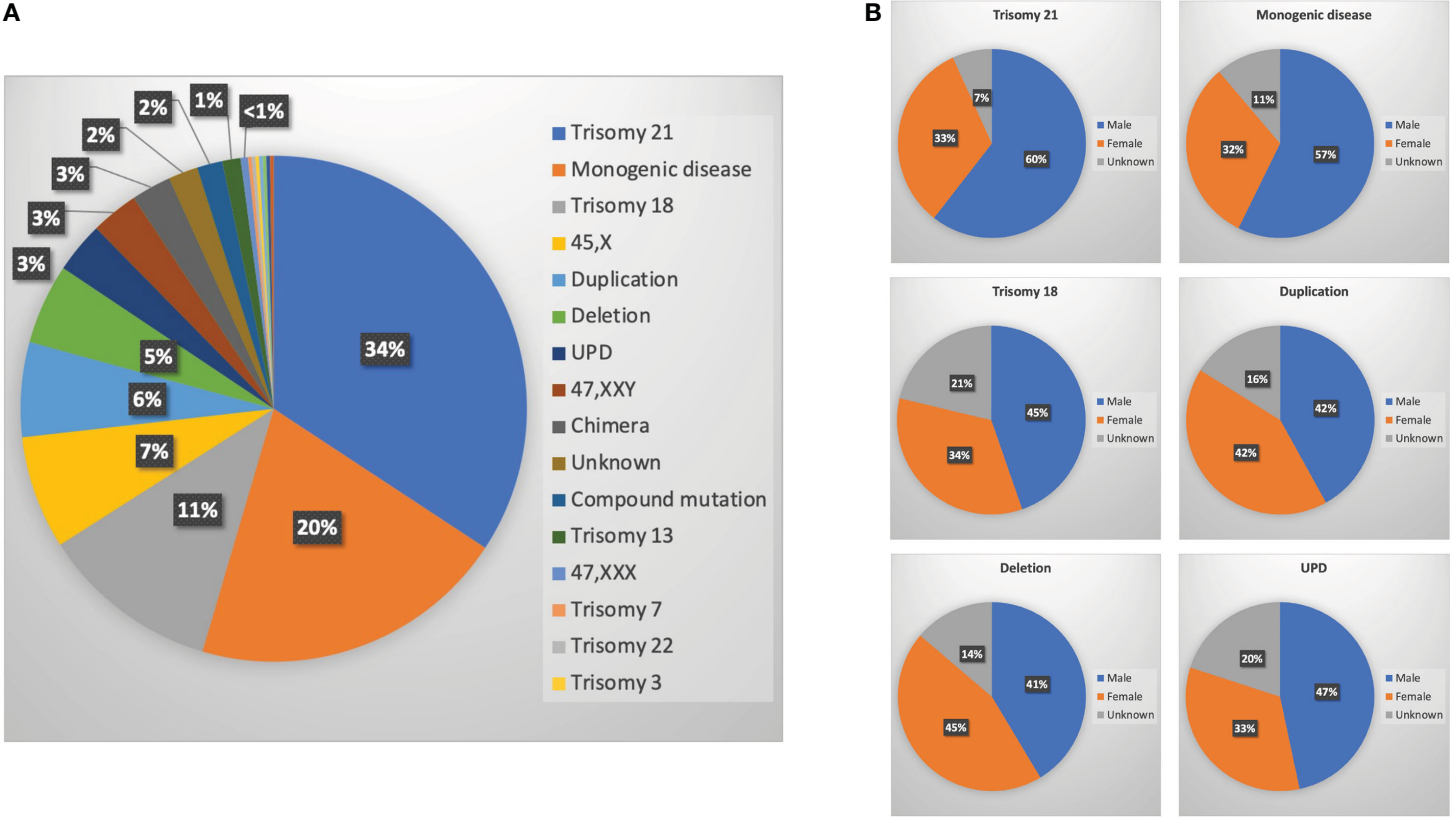
Genetic testing for malformed fetuses. **(A)** Ratio of different genetic abnormalities; **(B)** sex ratio of six main genetic abnormalities [trisomy 21, monogenic diseases, trisomy 18, duplications, deletions, and uniparental disomy (UPD)].

Overall, 725 of 1,661 cases had at least one genetic test performed, of which 296 (41%) were microarray or chromosomally normal and 429 (59%) were abnormal. No significant difference of sex ratio between chromosomally normal or abnormal cases was observed (**Supplementary Figure S2**). Among them, 147 cases had trisomy 21, 87 cases had a monogenic disease, 49 cases had trisomy 18, 31 cases had Turner’s syndrome, 26 had duplications, 22 had deletions, 14 had UPD, 13 had Klinefelter syndrome, 11 had chimeras, eight had an unknown genetic disorder (the genetic test was performed in another hospital with results not provided), seven had compound mutations (four microdeletions + microduplications; one microduplication + UPD, one monogenic disease + microdeletion, one microdeletion + balanced translocation), five had trisomy 13, one had trisomy 7, one had trisomy 22, one had trisomy 3, one had 47 XYY, one had inversion, one had a translocation, and one was triploid (**Figure 3A** and **Supplementary Table S1**). Among them, the highest proportion of cases was trisomy 21 with 34%, followed by monogenic disease and trisomy 18 at 20% and 11%, respectively (**Figure 3B**).

## Discussion

A total of 1,661 fetal malformation cases were included in the analysis. The sex ratio of all malformation fetuses was 1.446. All kinds of structural malformations were classified into 13 types. Cardiopulmonary had the highest proportion of all malformation types at 28%. In most malformation types, the sex ratios were similar with a total of 1.446. However, chest and abdomen and NT/Multy malformations had significantly higher proportions of males. Digestive system malformation had a significantly higher female ratio. Maternal age was associated with genetic factors and inversely associated with brain malformations. Genetic tests indicated that more males were found with trisomy 21, trisomy 18, and monogenetic diseases, but duplications, deletions, and UPD had a similar sex ratio of males and females. No significant differences were detected because of the limited case number.

Human sperm cells are produced by symmetrical meiosis carrying X and Y chromosomes at equal frequencies. Because ova bear only X chromosomes, the sex ratio should theoretically be exactly 1 (1). One study analyzed 139,704 Assisted Reproduction Technology (ART) embryos and found that the sex ratio of day 0 to day 6 embryos was close to 1.008 (5). However, the apparent sex ratio at birth, also known as the secondary sex ratio, was significantly deviated to male dominance, suggesting that a proportion of embryos were lost between conception and birth (6). Fetal malformation might be one important event of these losses. Several population-based studies have estimated the sex ratio of ultrasound detectable malformations. Data from 3,469 miscarriage cases in the USA from 1977 to 1981 showed a total sex ratio of 1.25 (7). Data from 28,965 live births with at least one major defect in the USA from 1968 to 1995 had a sex ratio of 1.45 (8). Another study from 1989 to 1997 showed that the sex ratio of those with structural congenital malformations was 1.22 (9). Data from the United Kingdom from 1990 to 2009 showed that the sex ratio of an overall risk of any congenital anomaly was 1.26 (10). The sex ratios of different malformation subtypes also varied. Urinary tract defects, gastrointestinal tract defects, and congenital hypertrophic pyloric stenosis were male dominant (8, 11, 12). Nervous system defects (except spina bifida without hydrocephaly (12)), endocrine system defects, congenital dislocation of the hip, and intrauterine growth restriction were female dominant during pregnancy (8, 11), as well as after birth (9). However, cases with skull congenital abnormalities (CAs), particularly craniosynostosis, had a male excess (13). Eighteen registries from 24 countries looking at 108,534 samples from Europe indicated a male excess of cardiac defects and omphalocele, as well as a female excess of neural tube defects and gastroschisis (14). Data from the North of England from 1985 to 2003 collected 12,795 eligible cases, where 40% of malformation subtypes were male dominant and 12% of unique subtypes were female dominant (15). A large population-based study included data from 25,952 cases in the USA between 1997 and 2009. It provided an accurate assessment of several malformations that had large differences between the sexes, such as craniosynostosis (2.12), cleft lip with cleft palate (2.01), and cleft lip without cleft palate (1.78). The lowest observed sex ratios (female preponderance) were for choanal atresia (0.45), cloacal exstrophy (0.46), and holoprosencephaly (0.64) (16). Although researching the sex ratios of fetus malformations has been of concern for some time, the relative study of this topic is limited, especially in Asian populations. Our data showed a similar trend to the previous data of male dominance for cardiopulmonary and CLP and female dominance for digestive system malformations. However, we also showed male dominance for hernia, omphalocele, and gastroschisis. Larger studies are also needed to determine if these observations are from racial differences. So far, all data suggested that the proportion of male in fetal malformation induced pregnancy loss was still over 50%. The bias in sex ratio from embryo to fetus remain to be studied from other aspects.

The sex ratio is also affected by environmental factors, biological events and ethics group diversity. Fukuda et al. analyzed the effects of the January 1995 earthquake in Kobe, Japan and found that the acute stress resulted in a female preponderance 9 months later (17). A female preponderance was also reported among a rural African society in 3,282 children born from 684 women who had an adverse nutritional status (18). Maternal exposure to electromagnetic fields has been associated with a reduction in male offspring (19). It was suggested that the increase in male stillborn rates was associated with a “deterioration” in unspecified environmental conditions. Additionally, social factors should not be underestimated. The sex ratio in Italy decreased from 1.05 to 1.00 after the Chernobyl nuclear incident (20). In addition, ethics groups could impact the sex ratio by shaping societal attitudes toward sex practices. Some ethics groups may have a particular preference for a certain sex. Also, some ethics groups may advocate for equal treatment of both male and female fetuses. For example, an estimated 6 million female fetuses may have been selectively aborted in India between 1980 and 2010 (21). China’s population censuses reported that the sex ratio was 1.076 in 1982, rose to 1.115 in 1990, then to 1.199 in 2000 and 1.212 (22).

In addition to the external environment, the maternal *in utero* environment can also play a role. For some biological events, sex-related differences can be the direct outcome of genetic and hormone effects, depending on gonadal differentiation. Examples of such events are the normal or abnormal development of genital organs and anomalies such as pyloric hypertrophy (23) and gastric teratoma (24) (which although unrelated to genital development are probably influenced by testosterone). Altered sex ratios have been found in patients with sacrococcygeal teratoma, where there is a predominance of females (25), as well as with transposition of the great arteries, where there are more males than females (16). Offspring of mothers with insulin-dependent diabetes mellitus have shown a lowered sex ratio (26), a higher sex ratio (27), or no effect whatsoever depending on the study (28). The excess of daughters born to congenital adrenal hyperplasia mothers is from the hormonal treatment of these women prior to conception (29).

Genetic testing revealed the relationship between abnormal fetuses and gender from a new perspective. The sex ratios of trisomy 13, trisomy 18, and trisomy 21 were 0.88 (*n* = 584), 0.9 (*n* = 1702), and 1.16 (*n* = 3154), respectively, but the mosaics 46/47, +21 was 0.83 (30). Other studies have indicated that trisomy 21 had a higher proportion of males with sex ratios of 1.30 and 1.36 (31). Genetic mechanisms of male predominance in trisomy 21 include joint segregation of chromosome 21 and the Y chromosome in spermatogenesis, and chromosome 21 nondisjunction during the second meiotic division of oogenesis was caused by Y chromosome–bearing spermatozoa (32). In trisomy 18, maternal meiosis II errors can cause male preponderance and meiosis I errors can cause a female preponderance (33).

The male predominance in humans suggests that there is an evolutionary adaptation to the higher newborn and infant mortality for males (34). This centers on the concept that more males need to be born to have enough survivors for the maintenance of the species. Although providing strong evidence in support of this theory has been difficult, the overall male preponderance in births has existed since birth records have been kept.

Even though we have collected data as comprehensively as possible within the past 10 years, there are still limitations to this single-center cross-sectional study. The location of the hospital may not be representative of the entire population, as the region can affect the results. Lifestyle, dietary habits, and environmental exposures can easily serve as confounding factors. The results of limited genetic testing and ultrasound diagnosis cannot accurately diagnose certain chromosomal changes of unknown structure. The study only mentions the association between maternal age and fetal malformations, but not causality, so further studies should focus on determining if the observed associations between sex, fetal malformations, and genetic factors are causally related.

## Conclusions

Sex differences in the number of fetal malformations are common, and the proportion of males is often higher in malformed fetuses. Cardiopulmonary cases are the most common malformation type. Brain malformations were inversely associated with maternal age, while genetic factors were positively correlated with maternal age. Hernia, omphalocele, gastroschisis, and NT/Multy malformations had significantly higher male proportions, while digestive system malformations had a higher proportion of female fetuses. Trisomy 21, trisomy 18, and monogenetic diseases had higher proportions of males, but duplications, deletions, and UPD had a similar sex ratio between males and females in the Asian population. Future research could focus on whether pregnancy loss prior to 12 weeks is a cause of the observed sex ratio bias.

## Data availability statement

The datasets presented in this article are not readily available because hospital data involves patients confidential information. Requests to access the datasets should be directed to mxz329@163.com.

## Author contributions

Y-PS planned the study, YS and MZ collected the data. MZ wrote the manuscript. All authors contributed to the article and approved the submitted version.
