# Supplementary material for: Gender bias in fetal malformations: A cross-sectional study in Asian populations
Source: Front Endocrinol (Lausanne). 2023 Mar 30;14:1146689. doi: 10.3389/fendo.2023.1146689 (PMC10101566; doi:10.3389/fendo.2023.1146689)
Supplement: Supplementary file 1 [file DataSheet_1.docx]

**Supplementary File**


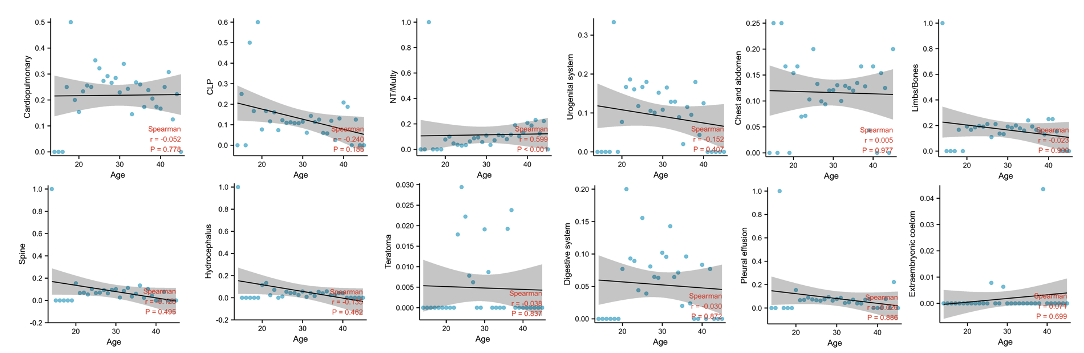


**Supplementary Figure 1** Malformations not associated with maternal age.


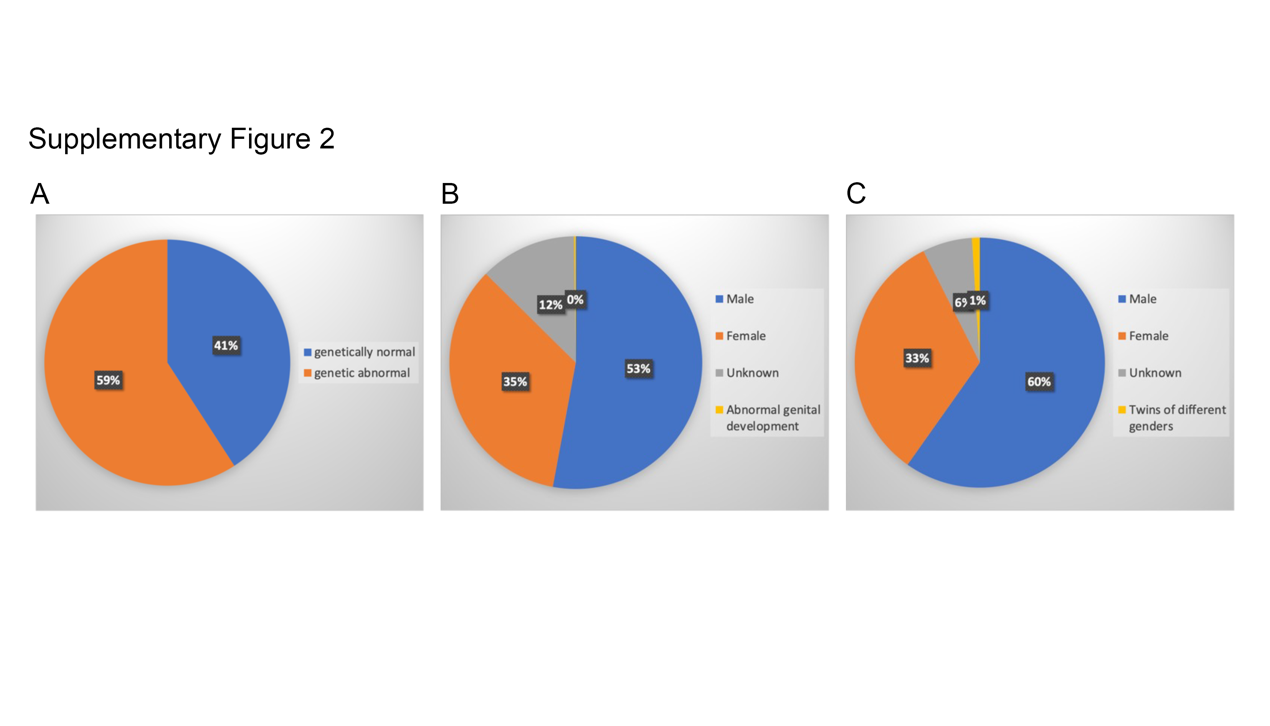


**Supplementary Figure 2** Sex ratio between chromosomally normal or abnormal cases. (A) The ratio of genetically normal and abnormal fetus in all malformation cases; (B) Sex ratio of genetically normal fetus; (C) Sex ratio of genetically abnormal fetus.

|  | Male | Female | Unknow or others | Total | Male/Female ratio | Correted *X2* | P |
| --- | --- | --- | --- | --- | --- | --- | --- |
| Trisomy 21 | 89 | 48 | 10 | 147 | 1.854 | 1.544 | 0.214 |
| Monogenic disease | 51 | 28 | 10 | 87 | 1.821 | 0.708 | 0.400 |
| Trisomy 18 | 21 | 16 | 10 | 49 | 1.313 | 0.014 | 0.905 |
| 45,X | / | / | / | 31 | / | / | / |
| Duplication | 13 | 13 | 5 | 26 | 1.000 | 0.542 | 0.462 |
| Deletion | 12 | 13 | 4 | 22 | 0.923 | 0.838 | 0.360 |
| UPD | 7 | 5 | 3 | 14 | 1.400 | 0.057 | 0.811 |
| 47,XXY | / | / | / | 13 | / | / | / |
| Chimera | 6 | 5 | 0 | 11 | 1.200 | 0.000 | 1.000 |
| Unknown | 6 | 2 | 2 | 8 | 3.000 | 0.303 | 0.582 |
| Compound mutation | 4 | 1 | 2 | 7 | 4.000 | 0.243 | 0.622 |
| Trisomy 13 | 2 | 2 | 1 | 5 | 1.000 | 0.019 | 0.890 |
| 47,XXX | / | / |  | 2 | / | / | / |
| Trisomy 7 | 1 | 0 | 0 | 1 | / | / | / |
| Trisomy 22 | 1 | 0 | 0 | 1 | / | / | / |
| Trisomy 3 | 0 | 0 | 1 | 1 | / | / | / |
| 47,XYY | / | / | / | 1 | / | / | / |
| Inversion | 0 | 0 | 1 | 1 | / | / | / |
| Translocation | 0 | 1 | 0 | 1 | / | / | / |
| Triploid | 0 | 1 | 0 | 1 | / | / | / |

**Supplementary Table 1** Sex ratio of 429 genetic abnormal fetuses (Chi-squared tests applied to categorical variables, corrected *X^2^*).
